# CD44 and Tumor-Derived Extracellular Vesicles (TEVs). Possible Gateway to Cancer Metastasis

**DOI:** 10.3390/ijms22031463

**Published:** 2021-02-02

**Authors:** Rafał Szatanek, Monika Baj-Krzyworzeka

**Affiliations:** Department of Clinical Immunology, Institute of Paediatrics, Jagiellonian University Medical College, 30-663 Kraków, Poland; mibaj@cyf-kr.edu.pl

**Keywords:** tumor-derived extracellular vesicles, extracellular vesicles, CD44, hyaluronan, cancer metastasis

## Abstract

Cancer metastasis, the final stage of tumor progression, is a complex process governed by the interplay of multiple types of cells and the tumor microenvironment. One of the aspects of this interplay involves the release of various factors by the tumor cells alone or by forcing other cells to do so. As a consequence of these actions, tumor cells are prepared in favorable conditions for their dissemination and spread to other sites/organs, which guarantees their escape from immunosurveillance and further progression. Tumor-derived extracellular vesicles (TEVs) represent a heterogeneous population of membrane-bound vesicles that are being actively released by different tumors. The array of proteins (i.e., receptors, cytokines, chemokines, etc.) and nucleic acids (i.e., mRNA, miR, etc.) that TEVs can transfer to other cells is often considered beneficial for the tumor’s survival and proliferation. One of the proteins that is associated with many different tumors as well as their TEVs is a cluster of differentiation 44 in its standard (CD44s) and variant (CD44v) form. This review covers the present information regarding the TEVs-mediated CD44s/CD44v transfer/interaction in the context of cancer metastasis. The content and the impact of the transferred cargo by this type of TEVs also are discussed with regards to tumor cell dissemination.

## 1. Introduction

The search for an effective target(s) in anti-tumor therapy has been ongoing, since the dawn of modern medicine. Despite many attempts at finding such targets, a surgical procedure followed by chemotherapy and/or radiotherapy still seems to be the best option to have a chance at eradicating tumors, however, the outcomes, very often, turn out to be unsatisfactory. The ability of tumors to ‘trick’ the immune system by becoming ‘invisible’, one way or another, is still a very serious challenge to the scientific community. The decades’ worth of research has provided one conclusion for certain; that the tumor-immune system interactions are not controlled by a single ‘switch’, but are rather governed by a very complex network of mutual, ongoing interactions resembling a constant battlefield. In appreciation of this complexity, the search and utilization of multiple targets simultaneously in an attempt to shut down tumor cell machinery is probably the best option for a successful anti-tumor strategy.

In over a decade now, the field of extracellular vesicles (EVs) has captured the attention of many groups engaged in tumor research. The ability of the tumor cells to release tumor-derived extracellular vesicles (TEVs) sheds new light on the tumor-immune system interactions [1,2]. This recently discovered form of intercellular communication has proven that tumor cells can modify the immune cells either directly, by transferring different factors (receptors, proteins, nucleic acids, etc.) using TEVs [3] or indirectly, by forcing the surrounding cells to release their respective EVs with appropriate ‘cargo’ supplementing tumor’s further progression [4]. These types of EVs/TEVs exchange seem to play a pivotal role in cancer metastasis; the final stage of tumor development, which proves too often terminal [3,5].

Another important player that is associated with cancer development/metastasis is a cluster of differentiation 44 (CD44) in its standard (CD44s) and variant (CD44v) form. The expression of CD44 has been elevated in many types of cancer [2], often localizing in tumor cells’ filopodia [6], suggesting its role in cellular motility; a prerequisite for cancer spreading. Moreover, due to the ability to sequester growth factors (i.e., HGF, FGF, EGF) at the cell surface and presenting them to tyrosine kinase receptor (RTK) complexes (i.e., c-Met, EGFR), this receptor has been associated with the activation of a number of signaling pathways involved in cell proliferation, adhesion, invasion and metabolic shift [7,8]. CD44 has also been detected on TEVs, and together with the TEVs content, is thought to be involved in ‘pre-metastatic’ niche formation by binding to hyaluronan (hyaluronic acid, HA) is preferred, distal organs [9,10].

This review discusses the involvement of CD44 in association with TEVs in cancer progression with the emphasis on ‘pre-metastatic’ niche formation and metastasis. It focuses on the modifications inflicted by this receptor upon delivery via TEVs to the target cells, due to its multifaceted role in intracellular signaling.

## 2. ‘Facts Check’—CD44 Receptor

CD44 is a receptor described as a single span transmembrane glycoprotein without kinase activity whose ubiquitous and constitutive expression has been observed on many different cells. It can also exist as an integral component of the extracellular matrix (ECM) or as a soluble protein found in body fluids [11]. Each of these protein forms plays biological roles, which may be shared or distinct [11].

**Figure 1:**
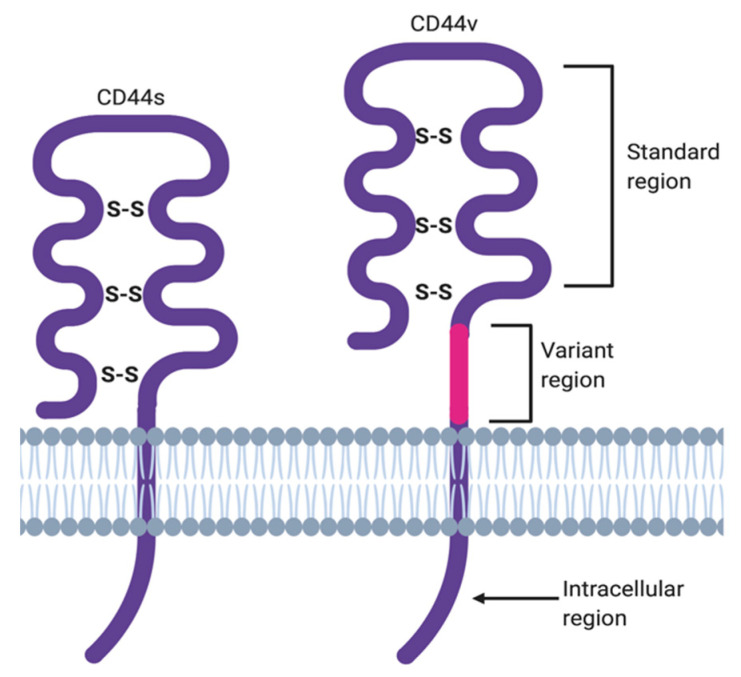
CD44 structure in its standard (CD44s) and variant (CD44v) forms.

**Figure 2:**
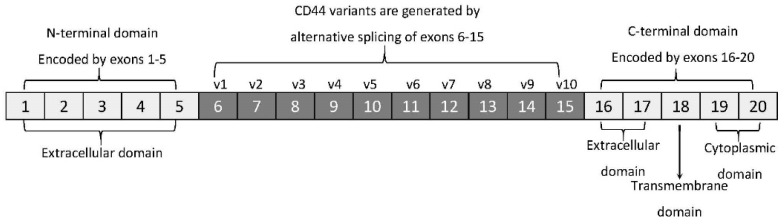
CD44 gene structure. CD44s form is encoded by exons 1–5 and 16–20 only, whereas CD44v form undergoes alternative splicing (exons 6–15).

The membrane form of CD44 is a receptor that binds ECM ligands, such as hyaluronan (HA), collagen, osteopontin, laminin, fibronectin, and others. The structure of CD44 in its standard (CD44s) and variant (CD44v) form is presented in Figure 1. CD44 is encoded by a single gene, which in humans is composed of 20 exons [12]. The schematic CD44 gene structure is presented in Figure 2. CD44s is composed of the homologous N-terminal and C-terminal domains only. The N-terminal domain is encoded by exons 1–5, whereas the C-terminal domain is encoded by exons 16–20. The molecular mass of CD44s is usually between 80–85 kDa. Multiple variant forms (CD44v, v1–v10) are generated by alternative splicing of exons 6–15, and are composed of both the N- and C-terminal domains and an additional variant domain(s). While CD44s is found in a wide variety of tissues, the CD44v isoform seems to have a much-restricted distribution and is observed to be present in keratinocytes, activated lymphocytes, macrophages, and some epithelial cells [13]. In both CD44 forms, the N-terminal domain binds the physiological ligands. CD44v isoforms are enriched in binding motifs, which promote the interactions with microenvironment components and may serve as co-receptors for growth factors, such as EGF and HGF [14]. Structural diversity of CD44 is also generated by posttranslational modification, such as phosphorylation, glycosylation, and attachment of glycosaminoglycans [11,15]. Resting cells show a low-affinity for binding CD44 ligands; however, cellular activation can induce a transition of CD44 to a high-affinity state that mediates binding to HA. The transition from the “inactive” low-affinity state to the “active” high-affinity state of CD44 can be induced by soluble factors, including cytokines [16,17]. Activation of CD44 leads to clustering and initiation of intracellular signaling pathways, such as ERK1, 2, AKT, FAK, Rho, Src, etc. (reviewed in [7]). Tumor cells express CD44 in a high-affinity state with the capacity for constitutive binding of HA [18]. The soluble and/or integral with ECM forms of CD44 are generated by metalloproteinase-inflicted (mainly MT1- and MT3-MMPs) shedding from a cell’s membrane [19]. The soluble CD44 protein can also be also produced by alternative splicing [20]. This form has been detected in the circulation and other body fluids, such as lymphatic, arthritic synovial, and bronchoalveolar fluids, as well as in cancer patients’ serum [21,22,23]. Soluble CD44 overexpression blocks cancer cell adhesion to HA [24] and is associated with aggressive growth and unfavorable prognosis for a patient.

## 3. Hyaluronan—The Major Ligand of CD44

**Figure 3:**
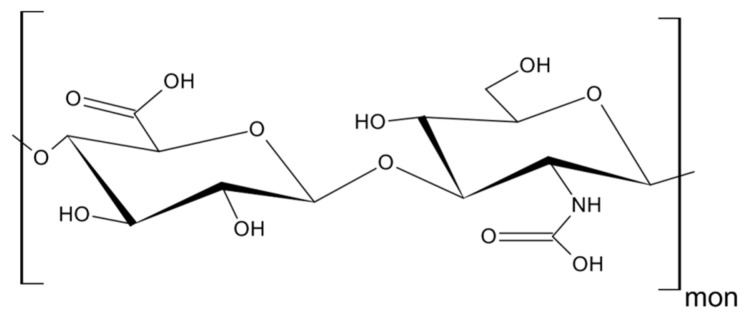
Structure of hyaluronan (HA).

As mentioned above, CD44 binds to a number of ligands; however, hyaluronan (hyaluronic acid, HA) is its most specific ligand that is recognizable by both standard and variant forms of this receptor [7]. HA is an anionic, non-sulfated polysaccharide that belongs to glycosaminoglycans. Its basic disaccharide monomer is a D-glucuronic acid linked with N-acetyl-D-glucosamine by glycosidic bonds (β1–4, β1–3). The schematic structure of HA is presented in Figure 3. The molecular weight of HA varies from 10^3^ to 10^7^ Da and depends on its tissue origin. In contrast to other glycosaminoglycans, HA is synthesized by three HA synthases (HAS 1–3) at the cell surface (not in Golgi apparatus) [25] and is released into the extracellular space. HA is a major component of ECM, which supports tissue organization and contributes to crucial processes, such as cell migration, proliferation, survival, etc. [26].

The downstream effects of HA binding depend on its molecular weight. Under normal circumstances, HA, in its “native state”, represents a high-molecular weight protein of about 1000 kDa [27] with anti-angiogenic, anti-inflammatory, and proliferation inhibiting activities [28]. On the other hand, HA in its ‘fragmented state’, with a molecular weight below 500 kDa, stimulates proliferation and migration of cells, induces an immune response, e.g., by increasing expression of inflammatory genes in human macrophages [27,29], the formation of immunosuppressive macrophages [30], death of activated T cells [31], and augments angiogenesis [28].

A number of studies have shown that HA is produced in abundance by malignant and stromal cells supporting the growth of tumors (breast [32], bladder [33], prostate [34] colorectal cancer [35]), and correlates with tumor aggressiveness in humans [30,35]. HA facilitates tumor cell proliferation, cell-to-cell adhesion and protects them from the immune cell response [36]. The balance between HA contradictory activities is governed by a complex system of HA synthases and hyaluronidases (Hyal), which regulate its degradation. Tumor cells have been reported to secrete excess amounts of hyaluronidases with the ability to cleave high molecular weight HA into smaller fragments (10–40 oligomers) [37], which, in turn, promoted CD44 cleavage [38]. Enhanced cleavage of CD44 was shown to facilitate tumor cell motility, which has been observed in many human tumors, including breast, colon, gastric, ovarian, lung, and others [37]. It has been shown that HA is involved in cancer cell, lymphocyte, and neutrophil motility in a CD44-dependent manner, however, the expression of this receptor alone was insufficient for chemotaxis towards HA [39,40]. Data on the binding efficiency of HA to CD44s and CD44v are conflicting. Bennett et al. reported that CD44s binds HA more efficiently than CD44v [41], however, other authors presented convincing data showing HA’s affinity to both receptors to be similar [42].

## 4. CD44-HA Interactions

There is quite an extensive number of surface receptors that recognize either the soluble or molecular form of HA. These include proteins, such as CD44, RHAMM, TSG-6/TNFIP6, Brevican, SHAP, LYVE1, TLRs, and others [26]. The RHAMM and TLR2,4 receptors preferentially bind smaller HA molecules (below 7 kDa), whereas CD44 requires at least six monosaccharides for the process to occur [reviewed in [43]. CD44 contains at least three HA binding sites on the hair loop (encoded by exon 2 and 5). CD44 molecules have been reported to be present in three functional stages: Constitutive binding of HA (most of the tumor cells), non-binding or non-binding until activated by physiological stimuli [7,44]. The affinity of HA to CD44 is cell-type dependent, regulated by glycosylation in the receptor’s extracellular domain and the phosphorylation of the serine residue in the cytoplasmic tail [45]. All forms of CD44 (standard or variants) can bind HA. In the case of immune cells, CD44 was described as the only receptor that has been demonstrated to bind HA [40].

**Figure 4:**
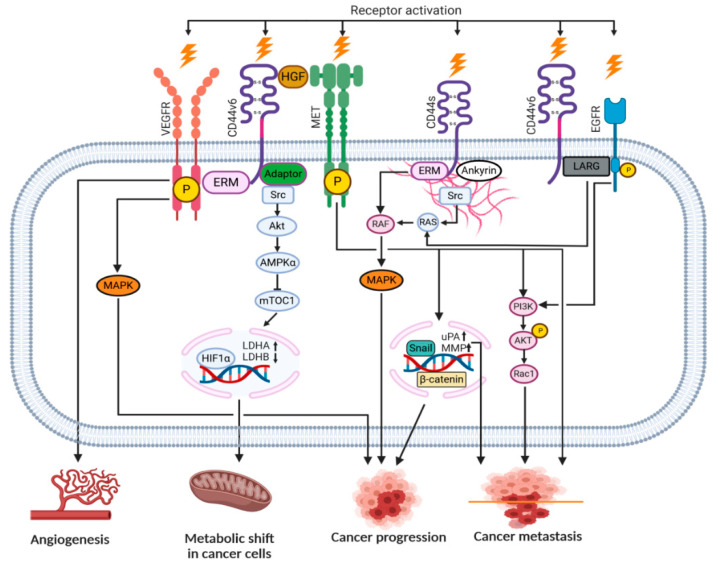
Signaling pathways engaging the CD44 receptor in cancer cells (according to [7] with own modifications).

Upon HA binding to the intercellular domain, CD44 undergoes conformational changes resulting in the recruitment of adaptor proteins (ERM, Src, etc.) to its intracellular domain, which, in turn, leads to the activation of a number of signaling pathways involved in cell proliferation, adhesion, migration, invasion and metabolic shift [8,46]. Table 1 summarizes all the currently known signal transduction mechanisms and their outcomes associated with CD44 molecule in cancer cells. Moreover, CD44v isoforms can also serve as co-receptors with the ability to sequester growth factors (i.e., HGF, FGF, EGF) at the cell surface and presenting them to tyrosine kinase receptor (RTK) complexes (i.e., c-Met, EGFR), which in turn, upon activation, can initiate an appropriate signaling pathway. Figure 4 depicts possible intercellular signaling mediated by the CD44 receptor in cancer cells.

CD44–HA interactions were defined as important for many types of cells. For example, macrophages exhibited preferential migration towards HA-enriched stromal structures in breast cancer [46]. It was also shown that small HA induced immunophenotypic maturation of human monocyte-derived dendritic cells and an increase in their pro-inflammatory cytokine (IL-1, TNF, and IL-12) production [47]. An elevated expression of CD44 has been reported on activated T cells [27]. The CD44-dependent adhesion mechanism is likely to be the most important for mobilizing the effector T cells at sites of infection and inflammation, because the expression of CD44 is upregulated whereas L-selectin (CD62L), the lymphocyte-expressed selectin, is downregulated in these cases [48]. Activation of CD44 influenced tumor cell metabolism by inducing Hypoxia-inducible factor 1α (HIF1α) binding to nuclear DNA to increase glycolysis, in turn, rendering a metabolic shift in cancer cells [49].

## 5. EMT and the Role of CD44 and TEVs

Prior to tumor cell dissemination, cancer cells in the primary tumor have to undergo epithelial-mesenchymal transition (EMT), which in the case of cancer is categorized as type III. EMT is a transdifferentiation process, crucial for tumor growth and progression, that allows cancer cells to detach from the basement of the tumor by undergoing changes necessary to reach mesenchymal phenotype and morphology. This step is characterized by HA overproduction [57], switching cadherin type from E to N, morphological changes (long shape, filopodia forming), and EVs secretion [6,58,59]. During the next phase of EMT, tumor cells undergo genetic and epigenetic changes favoring their dissemination, which are induced by specific signals that are still mostly undefined. However, factors, such as HGF, EGF, PDGF, and TGFβ, released by cells surrounding the tumor are considered to be very important in this process. There is also evidence, that transcription factors, such as Snail, Slug, and ZEB1, are crucial for the regulation of EMT [60].

Cho et al. reported the role of CD44 molecule in induction of EMT in SW480 colon cancer cell line [58]. In this case, activation of CD44 (e.g., ligand binding) initiated cytoskeleton changes (via ezrin-radixin-moesin connection) and MT1-MMP activation, which, in turn, promoted EGFR/Akt signaling pathway and downregulation of E-cadherin [58]. Others have also reported that CD44 interaction with HA induced malignant behavior of the tumor cells, which resulted in the initiation of several pathways, such as src/MAPK, PI3/Akt, and translocation of Snail/β-catenin to the nucleus [61]. In this auto-regulating loop, HA content on the surface of the tumor cells and between them is regulated by CD44 molecules, which induce HAS expression [6].

One of the modes aiding EMT involves EVs. EVs, in general, comprise a heterogeneous, membrane-bound population of vesicles that are released by different types of cells, including tumor surrounding cells (i.e., CAFs, macrophages, etc.) and cancer cells. EVs are found to be present in many biological fluids, including plasma, saliva, urine, seminal fluid, etc. [1], and are thought to play an important role in intercellular communication. Recently, TEVs were reported as a substantial contributor in EMT in cancer cells [62]. It was shown that treatment of noninvasive urothelial cells with TEVs isolated from invasive bladder cancer cell-conditioned media caused overexpression of, besides other factors, Snai1 in these cells, which resulted in their increased migration and invasion abilities [63]. In another study, human bronchial epithelial cells (HBECs) were exposed to either TEVs isolated from late-stage lung cancer patients or highly metastatic lung cancer cell-conditioned media [64]. In both cases, the exposed HBECs exhibited vimentin overexpression and EMT induction (upregulation of N-cadherin and the reduction of E-cadherin and ZO-1 expression), which later resulted in their elevated migratory, invasive, and proliferative capabilities [64]. Not surprisingly, EVs released by stromal cells can also induce EMT in cancer cells. EVs obtained from CD81^+^ CAFs subpopulation caused the autocrine release of Wnt11 from breast cancer cells (human breast MDA-MB-231 adenocarcinoma), resulting in their increased motility [65]. In a different study, enriched cargo of miRNA-21, -378e, and -143 was detected in CAF-derived EVs [66]. Treatment of breast cancer cells (BT549, MDA-MB-231, and T47D cell lines) with these EVs resulted in an increase in their capacity to form spheroids as portrayed by the expression of stem-cell and EMT markers [66]. Enhanced invasion and EMT promotion were also observed in tumor cells of the SKOV-3 and CAOV-3 cell lines that were exposed to CAF-derived EVs [67]. In this case, the isolated CAF-derived EVs carried elevated levels of TGFβ1, which promoted activation of the SMAD signaling pathway in these tumor cells [67].

CD44 has been reported to be present on TEVs derived from gastric cancer cell lines [6,68], ovarian [69], pancreatic [70,71], and primary mesenchymal cells [72]. The presence of HA-coated and CD44^+^ TEVs has been detected after their release by gastric cancer cells [6]. However, due to the limited knowledge regarding the molecular weight of HA carried by TEVs, it is difficult to predict their impact on EMT. For the most part, it is assumed that TEVs HA content potentially reflects the one from the cell of origin [72,73]; however, this issue needs further confirmation. TEVs carrying HA, oncogenes, miRNAs, and other ECM components can exert their impact by fusing with the cell membrane or by being engulfed by the target cells. In both cases, TEVs can influence target cells by delivering their content either directly (horizontal transfer) or indirectly through signaling pathway activation. It has been shown that TEVs carrying MMPs may remodel ECM [described in detail in [74,75,76] by downregulating E-cadherin expression, degrading ECM components, and upregulating MMP-9, which, all together, facilitated tumor dissemination [69]. Others observed that TEVs-delivered HA induced motility of CD44^+^ CAFs and cancer cells [reviewed in [77], and that CD44^+^ tumor cells present in the blood [77,78,79] exhibited characteristics of cancer stem cells. Expression of this receptor on these cells might results in their dissemination into niches (organotropism) that might be possibly prepared by HA-coated TEVs (see below).

## 6. Cancer Metastasis and TEVs

**Figure 5:**
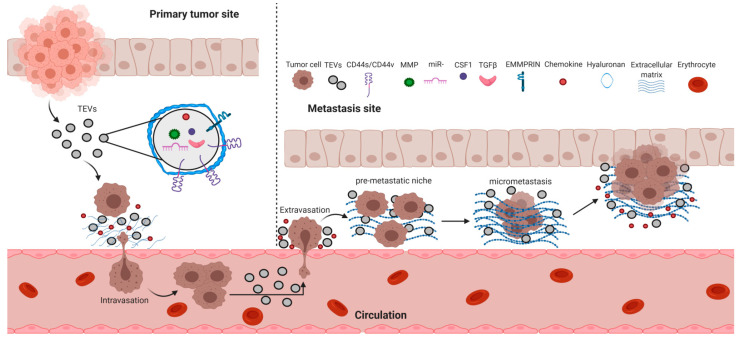
Schematic representation of the involvement of CD44/HA and TEVs in cancer transition from the primary tumor to metastasis.

Cancer metastasis is the final stage of tumor progression. It is defined by the arrival of primary tumor cells at other sites of the body, followed by their proliferation and formation of new tumors. The whole process of cancer metastasis can be divided into four main stages: Intravasation (from primary tumor sites to blood vessels), extravasation (from blood circulation to future metastasis sites), tumor latency, and formation of micrometastasis and macrometastasis [80]. Although it has been over a century since Steven Paget published his “seed and soil” hypothesis, stating that metastasizing cancer cells exhibit organotropism, the mechanisms underlying this phenomenon are still a mystery. Each stage of cancer metastasis seems to be governed by a number of different events (e.g., epithelial-mesenchymal transition, reverse mesenchymal-epithelial transition, extracellular matrix remodeling, etc.), however, the actual triggers and/or mediators of these events are still elusive. There is growing evidence that these ‘missing links’ between the stages comprising cancer metastasis might be filled by TEVs [81]. The overall cancer metastasis mechanism emphasizing TEVs involvement is graphically presented in Figure 5. Table 2 summarizes the content of the cargo carried by CD44s/CD44v-positive TEVs and depicts the impact these TEVs exert on different cellular processes.

### 6.1. Tumor Cell Intravasation

The concept of tumor cell intravasation involves the ability of cancer cells to invade into the normal tissue, often towards lymphatic or blood vessel, and then cross the endothelial barrier to enter circulation [87]. This process involves many different factors, including the presence of other cells in the tumor microenvironment, signaling molecules, proteases, and the proper environmental conditions at the tumor site and vasculature. Altogether, these factors play specific roles in allowing the cancer cell to invade through the basement membrane, adhere and pass through the endothelial cell junctions for circulation entry [88].

In the study involving large oncosomes (also referred as TEVs) isolated from the LNCaP prostate adenocarcinoma cell line, it was shown that they contained bioactive matrix MMP-2 and -9, key proteases involved in tumor cell invasion [89]. This finding allowed the authors to speculate that large oncosomes may cause degradation of the ECM and the release of endothelial permeabilization factors that facilitate intravasation [89]. MMP-14 has been demonstrated to be actively loaded into TEVs by the v-SNARE and VAMP3 proteins in amoeboid-like invasive tumor cell lines [71]. TEVs release by these tumor cells caused an increase in ECM degradation [71]. It was also reported that TEVs released by human renal cancer stem cells contained, besides other factors, MMP-2 and -9 mRNA, and that they were responsible for the preparation of favorable conditions for lung metastasis [90]. In another study, TEVs derived from a number of hypoxic lung cancer cell lines (CL1-5, NCI-H1437, -H1648, -H1792, and -H2087) carried miR-23a, which caused the suppression of prolyl hydroxylase 1 and 2 (PHD1 and 2) in endothelial cells leading to the accumulation of HIF-1α, known for its role in angiogenesis. Moreover, TEVs miR-23a also inhibited tight junction protein ZO-1, resulting in an increase in vascular permeability and cancer cell intravasation [91].

There is also growing evidence that CD44s/CD44v-TEVs may play a pivotal role in cancer cell intravasation. The importance of CD44v6 in the process of metastasis was first presented in the studies involving rats and highly metastatic BSp73ASML (ASML) cell line with a selective knockdown of the CD44v6 gene (CD44v6^kd^) [92,93]. Based on the obtained data, it was concluded that the CD44v6^kd^ tumor cells were unable to grow and that the rats remained tumor-free. However, the matrix obtained from wt ASML cell line sufficed for metastasis development. The separation of TEVs from this matrix revealed that they are mainly bound to HA, laminin, and collagen. These TEVs also contained high amounts of proteases and were able to degrade ECM proteins, a prerequisite of cancer cell intravasation [10,92].

Recruitment/transformation of a specific type of cells in the site of the primary tumor microenvironment enables successful cancer cell intravasation. A key feature in this process is the differentiation of stromal fibroblasts into myofibroblasts, which is associated with TGFβ and SMAD-dependent signaling [94,95]. There are numerous reports implying that tumor cells deliver TGFβ to fibroblasts by TEVs release, which initiates fibroblast differentiation [94,96]. It was also shown that transfer of MMP-inducer EMMPRIN by TEVs derived from lung cancer cells to fibroblasts led to MMP expression in the latter and ECM remodeling at the tumor site, thus, aiding tumor cell invasion [97].

Macrophages represent another type of cells that seems to play a major role in tumor cell intravasation [88]. Tumor cells can recruit and reprogram macrophages to support their growth and spread, which may well be facilitated by TEVs. One such example involves EGF/CSF1 paracrine loop between breast cancer cells and macrophages, where tumor cells secrete CSF1 to recruit macrophages, and in turn, macrophages release EGF to stimulate tumor cell invasion towards blood vessels [98]. Once at the endothelial barrier, proteases are being produced by both macrophages and tumor cells, enabling tumor entry into the blood vessel [99]. It has been shown that this direct interaction between the two types of cells is contact-dependent and results in the formation of tumor cell invadopodia allowing the tumor cell penetration through the tight endothelial junctions and bloodstream entry [100].

Cancer initiating cells (CIC) are believed to be a population of cells that are specifically engaged in metastasis. Invadopodia of these cells are crucial structures responsible for ECM degradation, and subsequent tumor cell migration and invasion [101,102,103,104,105]. CD44s/CD44v, being one of the CIC markers, is often associated with invadopodia. In colorectal carcinoma (CRC), binding of HA by CD44v6 causes the secretion of MMP-2 and -9, leading to changes in the ECM [106,107]. The presence of MMP-2 in this scenario may potentially result in the degradation of type IV collagen, a component of the basement membrane, causing tumor invasion [108]. Furthermore, the association of OPN with the ectodomain of the CD44v6 molecule leads to the phosphorylation of its intracellular domain, which, in turn, causes CCL5, CXCL12, and MMP-/-9 gene upregulation leading to the increase of CRC invasiveness [109]. There also seems to be an interplay between the CD44v6 receptor and the CCL12/CXCR4 signaling axis [110]. Treatment of non-metastatic CRC cells with CCL12 led to the acquisition of the CD44v6^+^ phenotype and the ability of these cells to form metastasis [111]. On the other hand, blocking of the CCL12/CXCR4 interaction caused reduced expression of CD44v6 in CRC stem cells [111]. In the presence of CCL12, there is an interaction between the intracellular domains of CD44v6 and CXCR4 receptors, whereas knockdown of CD44 impairs the CCL12-mediated CXCR4 signaling [112]. Altogether, it is reasonable to assume that the presence of CD44s/CD44v in the invadopodia of CIC is not a coincidence and that, together with other receptors, facilitates tumor cell metastasis with the emphasis on the initial phase of this process, namely, intravasation.

### 6.2. Tumor Cell Extravasation

The process of cellular passage from the bloodstream into the tissue is referred to as extravasation. The stages of this process seem to be conserved regardless of the type of cells involved, however, the most prominent cells engaged in this phenomenon are leukocytes, i.e., T lymphocytes, natural killer (NK), neutrophil granulocytes, and monocytes. Extravasation can be divided into three steps. The first step is characterized by the cells’ loose interaction through its surface receptors with vascular endothelium followed by their rolling along the blood vessel surface. The second step is characterized by a firm attachment of the cells to the endothelium, where a different set of receptors is being utilized. During the next, third step, referred to as diapedesis, the cells spread out on the endothelium and actively pass through the endothelial barrier [113]. The role of EVs in extravasation is still elusive, although, reports are emerging suggesting their involvement in this process. It has been reported that miR-181c delivered by TEVs obtained from brain metastatic cancer cells downregulated 3-phosphoinositide-dependent protein kinase-1 (PDPK1), causing cofilin-induced modulation of actin dynamics in the blood-brain barrier, which resulted in an increase in its permeability [114]. Furthermore, repeated exposure to these TEVs promoted preferential metastasis of breast cancer cells into the brain [114]. Zhou et al. showed that miR-105, a potent regulator of ZO-1, present in TEVs obtained from metastatic breast cancer cells, was able to completely destroy tight junctions in epithelial monolayers [115]. There are in vivo studies where anti-miR-105 treatment was employed resulted in suppressed brain metastasis [115]. Chemokines and their receptors, considered as important factors in tumor cell extravasation, were reported to be transported by TEVs. For example, elevated levels of SDF-1/CXCL12 and CXCR4 bearing TEVs were found to be present in the peripheral blood and bone marrow plasma samples of patients with acute myelogenous leukemia (AML) [116]. Moreover, these TEVs were able to transfer the CXCR4 receptor to AML-derived HL-60 cells, enhancing their migration to SDF-1 in vitro and increasing their homing to the bone marrow of irradiated NOD/SCID/beta2m (null) mice [116]. Chen T et al. also detected the presence of CCL2, CCL3, CCL4, CCL5, and CCL20 in TEVs generated by tumor cells undergoing heat stress [117]. Others reported that the release of CCL2 by colon carcinoma cells and its subsequent interaction with the CCR2 receptor present on endothelial cells resulted in vascular permeability [118]. Consequently, an increase in tumor cell extravasation was observed [118].

There are no direct reports pointing to the involvement of CD44/CD44v^+^ TEVs in extravasation of the tumor cells. However, CD44v6 expression in cells, such as macrophages, has been attributed to their ability to extravasate into tissue [119]. Since tumor cell intravasation and extravasation depend on the disruption of tight endothelial junctions, it is reasonable to speculate that once in circulation and under certain conditions, TEVs with appropriate content (i.e., CD44, HA, miR-181c, -105, CCL2, etc.) can disrupt vascular tight endothelial junctions enabling the docking and passage of disseminating cancer cells.

### 6.3. Tumor Latency

The original concept of tumor latency, defined as a time interval between the exposure to a cancer-causing factor until cancer diagnosis, has changed dramatically over time [120]. With the current knowledge on this topic, it has been proposed that tumor latency is a complex, multifactorial process that cannot be traced to one, single event. On the contrary, it is widely accepted that this process takes time and that exposure to a cancer-causing agent only accelerates it [120]. One of the factors that may have an impact on the acceleration of tumor latency is CD44. It has been shown that CD44^+^, and not CD44^-^, tumor cells sorted out from the primary mouse xenografts of non-small cell lung cancer (H1299) tumors exhibited shorter tumor latency in the subsequent tumor formations [121]. Analogically, in nasopharyngeal carcinoma, tumor cell xenografts of the HK1 cell line with high expressions of CD44 and EpCAM showed a more rapid growth than the ones where tumor cells’ expression of CD44 was diminished [122]. As with tumor cell extravasation, there is no evident data suggesting direct involvement of CD44^+^ EVs in tumor latency. Studies that undertake this phenomenon are required to fully understand their possible impact on this process.

### 6.4. Pre-Metastatic Niche Formation

The most studied aspect of metastasis where TEVs seem to play an important role involves pre-metastatic niche formation that occurs prior to macroscopic tumor cell invasion [123]. Pre-metastatic niche refers to the microenvironment, which is well prepared for tumor cells to colonize and disseminate to distant organ sites [124]. This process is multilateral, however, it has been proposed that several key components released by different types of cells are required for its occurrence. These components include secreted factors from tumor cells (VEGF, PIGF, S100A8/A9, etc.), bone marrow-derived cells (VLA-4, Id3), suppressive immune cells (HSF1, girdin, fibronectin, etc.), host stromal cells (TGF-β, TNF-α, G-CSF, etc.) and/or delivered by extracellular vesicles (MIF, OPN, microRNA-122, etc.) [125].

Among many different types of EVs taking part in metastasis, CD44s/CD44v-positive TEVs released by CICs are gaining considerable attention in the process of pre-metastatic niche formation. In the study by Jung T et al. [9], it was shown that the overexpression of CD44v6 promoted metastasis, which was confirmed by a selective knockdown of CD44v4-v7 (ASML^kd^) in the highly metastatic BSp73ASML cell line (ASML^wt^). The authors argued that the settlement of metastasizing tumor cells required the establishment of special niches in pre-metastatic organs, which was facilitated by the combination of soluble matrix and TEVs derived from these cells. They observed that CD44v6 played a central role in assembling the proper soluble matrix, which supported TEVs in the modulation of target cells in the pre-metastatic organ. The involvement of CD44 was also observed in the promotion of ovarian cancer invasion into the peritoneal cavity. In this case, the CD44^+^ TEVs released by the tumor cells of the HeyA8 ovarian cell line were able to transfer this receptor onto the human peritoneal mesothelial (HPMCs) cells. This, in turn, caused the reprogramming of the HPMCs cells to a more EMT phenotype, which led to an increase in ovarian cancer cell invasion and metastasis [69]. In another report concerning the degree of glioblastoma invasiveness, TEVs released by several glioblastoma cell lines were analyzed using mass spectrometry, and their specific marker profile was assessed [126]. The gathered data suggested a strong association between the TEVs CD44 expression and the level of invasiveness of the tested cell lines. The authors speculated that higher TEVs CD44 expression correlated with the more aggressive glioblastoma cell line, indicating their potential involvement in metastasis in this type of cancer. Another example of pre-metastatic niche formation where TEVs CD44 seems to play a crucial role involves pancreatic cancer progression [127]. The authors of this study demonstrated that CD44 not only interacted with integrin α6β4 and promoted tumor cell proliferation, migration, and invasion by regulating signaling pathways (Ras and ERK), but was also transferred by pancreatic TEVs to the liver cells- a common site of metastasis for this type of tumor. The authors argued that by interacting with the α6β4 integrin in the liver, CD44 generated a pre-metastatic milieu to facilitate specific organ metastasis by upregulating molecules, such as CD133, α-smooth muscle actin (α-SMA), and interleukin-6 (IL-6) in target liver cells [127].

## 7. Conclusions

The inability of the immune system to fully recognize tumor cells as foreign entities very often results in fatalities. The array of factors (cytokines, growth factors, receptor shedding, etc.) that tumor cells release into the surroundings to prevent or limit the immune response shows the type of arsenal at tumors’ disposal. In addition to these factors, tumor cells release TEVs, which not only modify the proximal tumor microenvironment, but also distal niches for future tumor cell dissemination. The release of TEVs with active receptors, such as CD44, with capabilities of physical interactions and intracellular signaling interference in other cells, has proven for tumors to be very effective in maintaining further progression/development. Organotropism of TEVs, such as CD44^+^TEVs, exemplifies the potential steps underlying the mechanisms involved in cancer metastasis; a critical step in determining the outcome of tumor treatment. Based on the accumulated information in this review, anti-tumor therapies aiming at the simultaneous blocking of both TEVs-directed CD44 loading and TEVs release might substantially undermine tumor spreading. This type of targeted interference, encompassing several critical points of cancer metastasis, presents a credible possibility of anti-tumor therapy that will bring humankind closer to the ultimate goal of defeating this disease.

## Figures and Tables

**Table 1:** Association of the CD44 molecule with signaling pathways in cancer cells.

	Signaling Pathway	Outcome	Reference
CD44s	ERM/MAPK	Cell division, proliferation	[50]
	PI3K/AKT/NFκB	Inflammatory cytokine release	[51]
	Migration of ICD fragment into the nucleus	Metastasis, survival	[52]
	RHAMM/cSrc/RAS	Migration	[53]
CD44v	ERM/FAK/Paxillin	Angiogenesis	[54]
	Src/AKT/HIF1α	Metabolic shift (glycolysis increase)	[49]
	Met/PI3K/AKT	Cell invasion	[55]
	Met/Snail/MMP	Cell division, proliferation	[56]

**Table 2:** Association of CD44s/CD44v-positive tumor-derived extracellular vesicles (TEV0s and their cargo content with the impact on cancer progression.

Type of Cancer	CD44 form Carried by TEVs	Cargo of TEVs	Role	Reference
Head and neck	CD44s, CD44v3	PD-L1, FasL, TGF-β, EGFR	Immunosupressive biomarker of disease	[82]
Bladder	CD44s	MUC-1, EMMPRIN, HLA I, integrins	disease monitoring,	[83]
Colon	CD44s	HA, EGFR, ADAM10	Cancer progression	[42,84,85]
	CD44v6	MMP2,-7,-9,-13,-14,-17, ADAM10, ADAM17, CD26, uPAR TIMP1, TIMP 2 CXCR4, MET, EpCAM, Tspan8, integrins	Motility and invasion promotion	[71]
Pancreas	CD44s, CD44v6	MET, EpCAM, integrin, CXCR4, uPAR, ADAM17, MMP2, 9, 7, 13, 14	Motility and invasion promotion	[71]
	CD44s, CD44v7–8	EpCAM, EMMPRIN, mRNA for VEGF, IL-8, HGF	interaction with monocytes	[70]
Ovarian	CD44s	n.a.	Invasion promotion	[69]
Breast	CD44s	n.a.	Drug resistance	[86]
Gastric	CD44s	HA	ECM interactions	[6]
Prostate	CD44s, CD44v8–10	CD44v8–10 mRNA	Drug resistance	[87]

## Data Availability

No new data were created or analyzed in this study. Data sharing is not applicable to this article.

## Abbreviations

TEVs	tumor-derived extracellular vesicles
EVs	extracellular vesicles
CD44	cluster of differentiation 44
CD44s	cluster of differentiation 44 standard isoform
CD44v	cluster of differentiation 44 variant isoform
HA	hyaluronan
HAS	hyaluronan synthase
ECM	extracellular matrix
EMT	epithelial-mesenchymal transformation
RTK	receptor tyrosine kinase
MMP	matrix matalloproteinase
CIC	cancer initiating cell
EMMPRIN	extracellular matrix metalloproteinase inducer
EGFR	epidermal growth factor receptor
CAFs	cancer associated fibroblasts
CRC	colorectal carcinoma
HIF1α	Hypoxia-inducible factor 1-alpha
ERM	ezrin-radixin-moesin
MAPK	mitogen-activated protein kinase
PI3K	phosphoinositide 3-kinase
AKT	alpha serine/threonine-protein kinase
NFκB	nuclear factor kappa-light-chain-enhancer of activated B cells
ICD	intracytoplasmic domain
RHAMM	receptor for hyaluronan-mediated motility
cSrc	proto-oncogene tyrosine-protein kinase Src
RAS	proto-oncogene protein p21
FAK	focal adhesion kinase
Met	hepatocyte growth factor receptor
Snail	zinc finger protein SNAI1

## References

[1] Yuana Y., Böing A.N., Grootemaat A.E., van der Pol E., Hau C.M., Cizmar P., Buhr E., Sturk A., Nieuwland R. (2015). Handling and storage of human body fluids for analysis of extracellular vesicles. J. Extracell. Vesicles.

[2] Zöller M. (2011). CD44: Can a cancer-initiating cell profit from an abundantly expressed molecule?. Nat. Rev. Cancer.

[3] Becker A., Thakur B.K., Weiss J.M., Kim H.S., Peinado H., Lyden D. (2016). Extracellular vesicles in cancer: Cell-to-cell mediators of metastasis. Cancer Cell.

[4] Park J.E., Dutta B., Tse S.W., Gupta N., Tan C.F., Low J.K., Yeoh K.W., Kon O.L., Tam J.P., Sze S.K. (2019). Hypoxia-induced tumor exosomes promote M2-like macrophage polarization of infiltrating myeloid cells and microRNA-mediated metabolic shift. Oncogene.

[5] Wu K., Xing F., Wu S.Y., Watabe K. (2017). Extracellular vesicles as emerging targets in cancer: Recent development from bench to bedside. Biochim. Biophys. Acta Rev. Cancer.

[6] Härkönen K., Oikari S., Kyykallio H., Capra J., Hakkola S., Ketola K., Thanigai Arasu U., Daaboul G., Malloy A., Oliveira C. (2019). CD44s assembles hyaluronan coat on filopodia and extracellular vesicles and induces tumorigenicity of MKN74 gastric carcinoma cells. Cells.

[7] Chen C., Zhao S., Karnad A., Freeman J.W. (2018). The biology and role of CD44 in cancer progression: Therapeutic implications. J. Hematol. Oncol..

[8] Ponta H., Sherman L., Herrlich P.A. (2003). CD44: From adhesion molecules to signalling regulators. Nat. Rev. Mol. Cell Biol..

[9] Jung T., Castellana D., Klingbeil P., Hernández I.C., Vitacolonna M., Orlicky D.J., Roffler S.R., Brodt P., Zöller M. (2009). CD44v6 dependence of premetastatic niche preparation by exosomes. Neoplasia.

[10] Mu W., Rana S., Zöller M. (2013). Host matrix modulation by tumor exosomes promotes motility and invasiveness. Neoplasia (US).

[11] Cichy J., Puré E. (2003). The liberation of CD44. J. Cell Biol..

[12] Yan Y., Zuo X., Wei D. (2015). Concise review: Emerging role of CD44 in cancer stem cells: A promising biomarker and therapeutic target. Stem Cells Transl. Med..

[13] Sneath R.J.S., Mangham D.C. (1998). The normal structure and function of CD44 and its role in neoplasia. J. Clin. Pathol. Mol. Pathol..

[14] Van Der Voort R., Taher T.E.I., Wielenga V.J.M., Spaargaren M., Prevo R., Smit L., David G., Hartmann G., Gherardi E., Pals S.T. (1999). Heparan sulfate-modified CD44 promotes hepatocyte growth factor/scatter factor-induced signal transduction through the receptor tyro sine kinase c-Met. J. Biol. Chem..

[15] Gao T., Wen T., Ge Y., Liu J., Yang L., Jiang Y., Dong X., Liu H., Yao J., An G. (2020). Disruption of Core 1-mediated O-glycosylation oppositely regulates CD44 expression in human colon cancer cells and tumor-derived exosomes. Biochem. Biophys. Res. Commun..

[16] Cichy J., Puré E. (2004). Cytokines regulate the affinity of soluble CD44 for hyaluronan. FEBS Lett..

[17] Cichy J., Puré E. (2000). Oncostatin M and transforming growth factor-β1 induce post-translational modification and hyaluronan binding to CD44 in lung-derived epithelial tumor cells. J. Biol. Chem..

[18] Naor D., Sionov R.V., Ish-Shalom D. (1997). CD44: Structure, function, and association with the malignant process. Adv. Cancer Res..

[19] Kajita M., Itoh Y., Chiba T., Mori H., Okada A., Kinoh H., Seiki M. (2001). Membrane-type 1 matrix metalloproteinase cleaves CD44 and promotes cell migration. J. Cell Biol..

[20] Yu Q., Toole B.P. (1996). A new alternatively spliced exon between v9 and v10 provides a molecular basis for synthesis of soluble CD44. J. Biol. Chem..

[21] Katoh S., McCarthy J.B., Kincade P.W. (1994). Characterization of soluble CD44 in the circulation of mice: Levels are affected by immune activity and tumor growth. J. Immunol..

[22] Katoh S., Taniguchi H., Matsubara Y., Matsumoto N., Fukushima K., Kadota J., Matsukura S., Kohno S. (1999). Overexpression of CD44 on alveolar eosinophils with high concentrations of soluble CD44 in bronchoalveolar lavage fluid in patients with eosinophilic pneumonia. Allergy Eur. J. Allergy Clin. Immunol..

[23] Shi M., Dennis K., Peschon J.J., Chandrasekaran R., Mikecz K. (2001). Antibody-induced shedding of CD44 from adherent cells is linked to the assembly of the cytoskeleton. J. Immunol..

[24] Päll T., Pink A., Kasak L., Turkina M., Anderson W., Valkna A., Kogerman P. (2011). Soluble CD44 interacts with intermediate filament protein vimentin on endothelial cell surface. PLoS ONE.

[25] Schwertfeger K.L., Cowman M.K., Telmer P.G., Turley E.A., McCarthy J.B. (2015). Hyaluronan, inflammation, and breast cancer progression. Front. Immunol..

[26] Toole B.P. (2009). Hyaluronan-CD44 interactions in cancer: Paradoxes and possibilities. Clin. Cancer Res..

[27] Jiang D., Liang J., Noble P.W. (2011). Hyaluronan as an immune regulator in human diseases. Physiol. Rev..

[28] Tolg C., McCarthy J.B., Yazdani A., Turley E.A. (2014). Hyaluronan and RHAMM in Wound Repair and the “cancerization” of Stromal Tissues. BioMed Res. Int..

[29] McKee C.M., Penno M.B., Cowman M., Burdick M.D., Strieter R.M., Bao C., Noble P.W. (1996). Hyaluronan (HA) fragments induce chemokine gene expression in alveolar macrophages: The role of HA size and CD44. J. Clin. Invest..

[30] Kuang D.M., Wu Y., Chen N., Cheng J., Zhuang S.M., Zheng L. (2007). Tumor-derived hyaluronan induces formation of immunosuppressive macrophages through transient early activation of monocytes. Blood.

[31] Ruffell B., Johnson P. (2008). Hyaluronan induces cell death in activated T cells through CD44. J. Immunol..

[32] Auvinen P., Rilla K., Tumelius R., Tammi M., Sironen R., Soini Y., Kosma V.M., Mannermaa A., Viikari J., Tammi R. (2014). Hyaluronan synthases (HAS1-3) in stromal and malignant cells correlate with breast cancer grade and predict patient survival. Breast Cancer Res. Treat..

[33] Lokeshwar V.B., Öbek C., Soloway M.S., Block N.L. (1997). Tumor-associated hyaluronic acid: A new sensitive and specific urine marker for bladder cancer. Cancer Res..

[34] Josefsson A., Adamo H., Hammarsten P., Granfors T., Stattin P., Egevad L., Laurent A.E., Wikström P., Bergh A. (2011). Prostate cancer increases hyaluronan in surrounding nonmalignant stroma, and this response is associated with tumor growth and an unfavorable outcome. Am. J. Pathol..

[35] Ropponen K., Tammi M., Parkkinen J., Eskelinen M., Tammi R., Lipponen P., Ågren U., Alhava E., Kosma V.M. (1998). Tumor cell-associated hyaluronan as an unfavorable prognostic factor in colorectal cancer. Cancer Res..

[36] McBride W.H., Bard J.B.L. (1979). Hyaluronidase-sensitive halos around adherent cells: Their role in blocking lymphocyte-mediated cytolysis. J. Exp. Med..

[37] Sugahara K.N., Hirata T., Hayasaka H., Stern R., Murai T., Miyasaka M. (2006). Tumor cells enhance their own CD44 cleavage and motility by generating hyaluronan fragments. J. Biol. Chem..

[38] Sugahara K.N., Murai T., Nishinakamura H., Kawashima H., Saya H., Miyasaka M. (2003). Hyaluronan oligosaccharides induce CD44 cleavage and promote cell migration in CD44-expressing tumor cells. J. Biol. Chem..

[39] Tzircotis G., Thorne R.F., Isacke C.M. (2005). Chemotaxis towards hyaluronan is dependent on CD44 expression and modulated by cell type variation in CD44-hyaluronan binding. J. Cell Sci..

[40] Lee-Sayer S.S.M., Dong Y., Arif A.A., Olsson M., Brown K.L., Johnson P. (2015). The where, when, how and why of hyaluronan binding by immune cells. Front. Immunol..

[41] Bennett K.L., Modrell B., Greenfield B., Bartolazzi A., Stamenkovic I., Peach R., Jackson D.G., Spring F., Aruffo A. (1995). Regulation of CD44 binding to hyaluronan by glycosylation of variably spliced exons. J. Cell Biol..

[42] Raman P.S., Alves C.S., Wirtz D., Konstantopoulos K. (2012). Distinct kinetic and molecular requirements govern CD44 binding to hyaluronan versus fibrin(ogen). Biophys. J..

[43] Campo G.M., Avenoso A., Micali A., Nastasi G., Squadrito F., Altavilla D., Bitto A., Polito F., Rinaldi M.G., Calatroni A. (2010). High-molecular weight hyaluronan reduced renal PKC activation in genetically diabetic mice. Biochim. Biophys. Acta Mol. Basis Dis..

[44] Lesley J., Hascall V.C., Tammi M., Hyman R. (2000). Hyaluronan binding by cell surface CD44. J. Biol. Chem..

[45] Misra S., Hascall V.C., Markwald R.R., Ghatak S. (2015). Interactions between hyaluronan and its receptors (CD44, RHAMM) regulate the activities of inflammation and cancer. Front. Immunol..

[46] Kobayashi N., Miyoshi S., Mikami T., Koyama H., Kitazawa M., Takeoka M., Sano K., Amano J., Isogai Z., Niida S. (2010). Hyaluronan deficiency in tumor stroma impairs macrophage trafficking and tumor neovascularization. Cancer Res..

[47] Termeer C., Sleeman J.P., Simon J.C. (2003). Hyaluronan—Magic glue for the regulation of the immune response?. Trends Immunol..

[48] Baaten B.J.G., Li C.R., Bradley L.M. (2010). Multifaceted regulation of T cells by CD44. Commun. Integr. Biol..

[49] Nam K., Oh S., Shin I. (2016). Ablation of CD44 induces glycolysis-to-oxidative phosphorylation transition via modulation of the c-Src-Akt-LKB1-AMPK pathway. Biochem. J..

[50] Nam K.S., Oh S., Lee K.M., Yoo S.A., Shin I. (2015). CD44 regulates cell proliferation, migration, and invasion via modulation of c-Src transcription in human breast cancer cells. Cell. Signal..

[51] Lokeshwar V.B., Mirza S., Jordan A. (2014). Targeting hyaluronic acid family for cancer chemoprevention and therapy. Adv. Cancer Res..

[52] Senbanjo L.T., Chellaiah M.A. (2017). CD44: A multifunctional cell surface adhesion receptor is a regulator of progression and metastasis of cancer cells. Front. Cell Dev. Biol..

[53] Nikitovic D., Kouvidi K., Karamanos N.K., Tzanakakis G.N. (2013). The roles of hyaluronan/RHAMM/CD44 and their respective interactions along the insidious pathways of fibrosarcoma progression. BioMed Res. Int..

[54] Orian-Rousseau V., Morrison H., Matzke A., Kastilan T., Pace G., Herrlich P., Ponta H. (2007). Hepatocyte growth factor-induced Ras activation requires ERM proteins linked to both CD44v6 and F-actin. Mol. Biol. Cell.

[55] Orian-Rousseau V., Chen L., Sleeman J.P., Herrlich P., Ponta H. (2002). CD44 is required for two consecutive steps in HGF/c-Met signaling. Genes Dev..

[56] Reinke L.M., Xu Y., Cheng C. (2012). Snail represses the splicing regulator epithelial splicing regulatory protein 1 to promote epithelial-mesenchymal transition. J. Biol. Chem..

[57] Baj-Krzyworzeka M., Weglarczyk K., Szatanek R., Mytar B., Baran J., Siedlar M. (2019). The role of CD44H molecule in the interactions between human monocytes and pancreatic adenocarcinoma-derived microvesicles. Folia Histochem. Cytobiol..

[58] Cho S.H., Park Y.S., Kim H.J., Kim C.H., Lim S.W., Huh J.W., Lee J.H., Kim H.R. (2012). CD44 enhances the epithelial-mesenchymal transition in association with colon cancer invasion. Int. J. Oncol..

[59] Toole B.P., Zoltan-Jones A., Misra S., Ghatak S. (2005). Hyaluronan: A critical component of epithelial-mesenchymal and epithelial-carcinoma transitions. Cells Tissues Organs.

[60] Kletukhina S., Neustroeva O., James V., Rizvanov A., Gomzikova M. (2019). Role of mesenchymal stem cell-derived extracellular vesicles in epithelial-mesenchymal transition. Int. J. Mol. Sci..

[61] Liu M., Tolg C., Turley E. (2019). Dissecting the dual nature of hyaluronan in the tumor microenvironment. Front. Immunol..

[62] Gopal S.K., Greening D.W., Rai A., Chen M., Xu R., Shafiq A., Mathias R.A., Zhu H.J., Simpson R.J. (2017). Extracellular vesicles: Their role in cancer biology and epithelial-mesenchymal transition. Biochem. J..

[63] Franzen C.A., Blackwell R.H., Todorovic V., Greco K.A., Foreman K.E., Flanigan R.C., Kuo P.C., Gupta G.N. (2015). Urothelial cells undergo epithelial-to-mesenchymal transition after exposure to muscle invasive bladder cancer exosomes. Oncogenesis.

[64] Rahman M.A., Barger J.F., Lovat F., Gao M., Otterson G.A., Nana-Sinkam P. (2016). Lung cancer exosomes as drivers of epithelial mesenchymal transition. Oncotarget.

[65] Luga V., Zhang L., Viloria-Petit A.M., Ogunjimi A.A., Inanlou M.R., Chiu E., Buchanan M., Hosein A.N., Basik M., Wrana J.L. (2012). Exosomes mediate stromal mobilization of autocrine Wnt-PCP signaling in breast cancer cell migration. Cell.

[66] Donnarumma E., Fiore D., Nappa M., Roscigno G., Adamo A., Iaboni M., Russo V., Affinito A., Puoti I., Quintavalle C. (2017). Cancer-associated fibroblasts release exosomal microRNAs that dictate an aggressive phenotype in breast cancer. Oncotarget.

[67] Li W., Zhang X., Wang J., Li M., Cao C., Tan J., Ma D., Gao Q. (2017). TGFβ1 in fibroblasts-derived exosomes promotes epithelialmesenchymal transition of ovarian cancer cells. Oncotarget.

[68] Szatanek R., Weglarczyk K., Stec M., Baran J., Parlinska-Wojtan M., Siedlar M., Baj-Krzyworzeka M. (2019). Autologous tumor-derived microvesicles influence gene expression profiles and enhance protumorigenic chemotactic potential, signal transduction and cellular respiration in gastric cancer cells. Int. J. Oncol..

[69] Nakamura K., Sawada K., Kinose Y., Yoshimura A., Toda A., Nakatsuka E., Hashimoto K., Mabuchi S., Morishige K.I., Kurachi H. (2017). Exosomes promote ovarian cancer cell invasion through transfer of CD44 to peritoneal mesothelial cells. Mol. Cancer Res..

[70] Baj-Krzyworzeka M., Szatanek R., Wȩglarczyk K., Baran J., Urbanowicz B., Brański P., Ratajczak M.Z., Zembala M. (2006). Tumour-derived microvesicles carry several surface determinants and mRNA of tumour cells and transfer some of these determinants to monocytes. Cancer Immunol. Immunother..

[71] Wang Z., von Au A., Schnölzer M., Hackert T., Zöller M. (2016). CD44v6-competent tumor exosomes promote motility, invasion and cancer-initiating cell marker expression in pancreatic and colorectal cancer cells. Oncotarget.

[72] Koistinen V., Härkönen K., Kärnä R., Arasu U.T., Oikari S., Rilla K. (2017). EMT induced by EGF and wounding activates hyaluronan synthesis machinery and EV shedding in rat primary mesothelial cells. Matrix Biol..

[73] Lenart M., Rutkowska-Zapala M., Baj-Krzyworzeka M., Szatanek R., W?glarczyk K., Smallie T., Ziegler-Heitbrock L., Zembala M., Siedlar M. (2017). Hyaluronan carried by tumor-derived microvesicles induces IL-10 production in classical (CD14^++^CD16^−^) monocytes via PI3K/Akt/mTOR-dependent signalling pathway. Immunobiology.

[74] Fares J., Fares M.Y., Khachfe H.H., Salhab H.A., Fares Y. (2020). Molecular principles of metastasis: A hallmark of cancer revisited. Signal Transduct. Target. Ther..

[75] Taraboletti G., D’Ascenzo S., Borsotti P., Giavazzi R., Pavan A., Dolo V. (2002). Shedding of the matrix metalloproteinases MMP-2, MMP-9, and MT1-MMP as membrane vesicle-associated components by endothelial cells. Am. J. Pathol..

[76] Dolo V., Ginestra A., Cassarà D., Violini S., Lucania G., Torrisi M.R., Nagase H., Canevari S., Pavan A., Vittorelli M.L. (1998). Selective localization of matrix metalloproteinase 9, β 1 integrins, and human lymphocyte antigen class I molecules on membrane vesicles shed by 8701-BC breast carcinoma cells. Cancer Res..

[77] McCarthy J.B., El-Ashry D., Turley E.A. (2018). Hyaluronan, cancer-associated fibroblasts and the tumor microenvironment in malignant progression. Front. Cell Dev. Biol..

[78] Szczepanik A., Sierzega M., Drabik G., Pituch-Noworolska A., Kołodziejczyk P., Zembala M. (2019). CD44^+^ cytokeratin-positive tumor cells in blood and bone marrow are associated with poor prognosis of patients with gastric cancer. Gastric Cancer.

[79] Pituch-Noworolska A., Wiȩckiewicz J., Krzeszowiak A., Stachura J., Ruggiero I., Gawlicka M., Szczepanik A., Karcz D., Popiela T., Zembala M. (1998). Evaluation of circulating tumour cells expressing CD44 variants in the blood of gastric cancer patients by flow cytometry. Anticancer Res..

[80] Tian W., Liu S., Li B. (2019). Potential role of exosomes in cancer metastasis. BioMed Res. Int..

[81] Hoshino A., Costa-Silva B., Shen T.L., Rodrigues G., Hashimoto A., Tesic Mark M., Molina H., Kohsaka S., Di Giannatale A., Ceder S. (2015). Tumour exosome integrins determine organotropic metastasis. Nature.

[82] Theodoraki M.N., Matsumoto A., Beccard I., Hoffmann T.K., Whiteside T.L. (2020). CD44v3 protein-carrying tumor-derived exosomes in HNSCC patients’ plasma as potential noninvasive biomarkers of disease activity. Oncoimmunology.

[83] Welton J.L., Khanna S., Giles P.J., Brennan P., Brewis I.A., Staffurth J., Mason M.D., Clayton A. (2010). Proteomics analysis of bladder cancer exosomes. Mol. Cell. Proteom..

[84] Choi D.S., Choi D.Y., Hong B.S., Jang S.C., Kim D.K., Lee J., Kim Y.K., Kim K.P., Gho Y.S. (2012). Quantitative proteomics of extracellular vesicles derived from human primary and metastatic colorectal cancer cells. J. Extracell. Vesicles.

[85] Purushothaman A., Bandari S.K., Chandrashekar D.S., Jones R.J., Lee H.C., Weber D.M., Orlowski R.Z. (2017). Chondroitin sulfate proteoglycan serglycin influences protein cargo loading and functions of tumor-derived exosomes. Oncotarget.

[86] Wang X., Cheng K., Zhang G., Jia Z., Yu Y., Guo J., Hua Y., Guo F., Li X., Zou W. (2020). Enrichment of CD44 in exosomes from breast cancer cells treated with doxorubicin promotes chemoresistance. Front. Oncol..

[87] Kato T., Mizutani K., Kawakami K., Fujita Y., Ehara H., Ito M. (2020). CD44v8-10 mRNA contained in serum exosomes as a diagnostic marker for docetaxel resistance in prostate cancer patients. Heliyon.

[88] Chiang S.P.H., Cabrera R.M., Segall J.E. (2016). Tumor cell intravasation. Am. J. Physiol. Cell Physiol..

[89] Di Vizio D., Morello M., Dudley A.C., Schow P.W., Adam R.M., Morley S., Mulholland D., Rotinen M., Hager M.H., Insabato L. (2012). Large oncosomes in human prostate cancer tissues and in the circulation of mice with metastatic disease. Am. J. Pathol..

[90] Grange C., Tapparo M., Collino F., Vitillo L., Damasco C., Deregibus M.C., Tetta C., Bussolati B., Camussi G. (2011). Microvesicles released from human renal cancer stem cells stimulate angiogenesis and formation of lung premetastatic niche. Cancer Res..

[91] Hsu Y.L., Hung J.Y., Chang W.A., Lin Y.S., Pan Y.C., Tsai P.H., Wu C.Y., Kuo P.L. (2017). Hypoxic lung cancer-secreted exosomal MIR-23a increased angiogenesis and vascular permeability by targeting prolyl hydroxylase and tight junction protein ZO-1. Oncogene.

[92] Klingbeil P., Marhaba R., Jung T., Kirmse R., Ludwig T., Zöller M. (2009). CD44 variant isoforms promote metastasis formation by a tumor cell-matrix cross-talk that supports adhesion and apoptosis resistance. Mol. Cancer Res..

[93] Jung T., Gross W., Zöller M. (2011). CD44v6 coordinates tumor matrix-triggered motility and apoptosis resistance. J. Biol. Chem..

[94] Webber J., Steadman R., Mason M.D., Tabi Z., Clayton A. (2010). Cancer exosomes trigger fibroblast to myofibroblast differentiation. Cancer Res..

[95] Webber J.P., Spary L.K., Sanders A.J., Chowdhury R., Jiang W.G., Steadman R., Wymant J., Jones A.T., Kynaston H., Mason M.D. (2015). Differentiation of tumour-promoting stromal myofibroblasts by cancer exosomes. Oncogene.

[96] Yen E.Y., Miaw S.C., Yu J.S., Lai I.R. (2017). Exosomal TGF-β1 is correlated with lymphatic metastasis of gastric cancers. Am. J. Cancer Res..

[97] Sidhu S.S., Mengistab A.T., Tauscher A.N., LaVail J., Basbaum C. (2004). The microvesicle as a vehicle for EMMPRin in tumor-stromal interactions. Oncogene.

[98] Goswami S., Sahai E., Wyckoff J.B., Cammer M., Cox D., Pixley F.J., Stanley E.R., Segall J.E., Condeelis J.S. (2005). Macrophages promote the invasion of breast carcinoma cells via a colony-stimulating factor-1/epidermal growth factor paracrine loop. Cancer Res..

[99] Wyckoff J.B., Wang Y., Lin E.Y., Li J.F., Goswami S., Stanley E.R., Segall J.E., Pollard J.W., Condeelis J. (2007). Direct visualization of macrophage-assisted tumor cell intravasation in mammary tumors. Cancer Res..

[100] Roh-Johnson M., Bravo-Cordero J.J., Patsialou A., Sharma V.P., Guo P., Liu H., Hodgson L., Condeelis J. (2014). Macrophage contact induces RhoA GTPase signaling to trigger tumor cell intravasation. Oncogene.

[101] Courtneidge S.A., Azucena E.F., Pass I., Seals D.F., Tesfay L. (2005). The SRC substrate Tks5, podosomes (Invadopodia), and cancer cell invasion. Cold Spring Harbor Symposia on Quantitative Biology.

[102] Eckert M.A., Lwin T.M., Chang A.T., Kim J., Danis E., Ohno-Machado L., Yang J. (2011). Twist1-Induced invadopodia formation promotes tumor metastasis. Cancer Cell.

[103] Paz H., Pathak N., Yang J. (2014). Invading one step at a time: The role of invadopodia in tumor metastasis. Oncogene.

[104] Seals D.F., Azucena E.F., Pass I., Tesfay L., Gordon R., Woodrow M., Resau J.H., Courtneidge S.A. (2005). The adaptor protein Tks5/Fish is required for podosome formation and function, and for the protease-driven invasion of cancer cells. Cancer Cell.

[105] Li C.M.C., Chen G., Dayton T.L., Kim-Kiselak C., Hoersch S., Whittaker C.A., Bronson R.T., Beer D.G., Winslow M.M., Jacks T. (2013). Differential Tks5 isoform expression contributes to metastatic invasion of lung adenocarcinoma. Genes Dev..

[106] Miletti-González K.E., Murphy K., Kumaran M.N., Ravindranath A.K., Wernyj R.P., Kaur S., Miles G.D., Lim E., Chan R., Chekmareva M. (2012). Identification of function for CD44 intracytoplasmic domain (CD44-ICD): Modulation of matrix metalloproteinase 9 (MMP-9) transcription via novel promoter response element. J. Biol. Chem..

[107] Ma D.-M., Luo D.-X., Zhang J. (2016). SDF-1/CXCR7 axis regulates the proliferation, invasion, adhesion, and angiogenesis of gastric cancer cells. World J. Surg. Oncol..

[108] Mook O.R.F., Frederiks W.M., Van Noorden C.J.F. (2004). The role of gelatinases in colorectal cancer progression and metastasis. Biochim. Biophys. Acta Rev. Cancer.

[109] Mi Z., Bhattacharya S.D., Kim V.M., Guo H., Talbotq L.J., Kuo P.C. (2011). Osteopontin promotes CCL5-mesenchymal stromal cell-mediated breast cancer metastasis. Carcinogenesis.

[110] Ma L., Dong L., Chang P. (2019). CD44v6 engages in colorectal cancer progression. Cell Death Dis..

[111] Todaro M., Gaggianesi M., Catalano V., Benfante A., Iovino F., Biffoni M., Apuzzo T., Sperduti I., Volpe S., Cocorullo G. (2014). CD44v6 is a marker of constitutive and reprogrammed cancer stem cells driving colon cancer metastasis. Cell Stem Cell.

[112] Orian-Rousseau V., Sleeman J. (2014). CD44 is a multidomain signaling platform that integrates extracellular matrix cues with growth factor and cytokine signals. Adv. Cancer Res..

[113] Strell C., Entschladen F. (2008). Cell communication and signaling extravasation of leukocytes in comparison to tumor cells. Cell Commun. Signal..

[114] Tominaga N., Kosaka N., Ono M., Katsuda T., Yoshioka Y., Tamura K., Lötvall J., Nakagama H., Ochiya T. (2015). Brain metastatic cancer cells release microRNA-181c-containing extracellular vesicles capable of destructing blood-brain barrier. Nat. Commun..

[115] Zhou W., Fong M.Y., Min Y., Somlo G., Liu L., Palomares M.R., Yu Y., Chow A., O’Connor S.T.F., Chin A.R. (2014). Cancer-Secreted miR-105 destroys vascular endothelial barriers to promote metastasis. Cancer Cell.

[116] Kalinkovich A., Tavor S., Avigdor A., Kahn J., Brill A., Petit I., Goichberg P., Tesio M., Netzer N., Naparstek E. (2006). Functional CXCR4-expressing microparticles and SDF-1 correlate with circulating acute myelogenous leukemia cells. Cancer Res..

[117] Chen T., Guo J., Yang M., Zhu X., Cao X. (2011). Chemokine-containing exosomes are released from heat-stressed tumor cells via lipid raft-dependent pathway and act as efficient tumor vaccine. J. Immunol..

[118] Wolf M.J., Hoos A., Bauer J., Boettcher S., Knust M., Weber A., Simonavicius N., Schneider C., Lang M., Stürzl M. (2012). Endothelial CCR2 signaling induced by colon carcinoma cells enables extravasation via the JAK2-Stat5 and p38MAPK pathway. Cancer Cell.

[119] Braumüller H., Gansauge S., Ramadani M., Gansauge F. (2000). CD44v6 cell surface expression is a common feature of macrophages and macrophage-like cells—Implication for a natural macrophage extravasation mechanism mimicked by tumor cells. FEBS Lett..

[120] Abecasis M., Cross N.C.P., Brito M., Ferreira I., Sakamoto K.M., Hijiya N., Score J., Gale R.P. (2020). Is cancer latency an outdated concept? Lessons from chronic myeloid leukemia. Leukemia.

[121] Leung E.L.H., Fiscus R.R., Tung J.W., Tin V.P.C., Cheng L.C., Sihoe A.D.L., Fink L.M., Ma Y., Wong M.P. (2010). Non-small cell lung cancer cells expressing CD44 are enriched for stem cell-like properties. PLoS ONE.

[122] Hoe S.L.L., Tan L.P., Abdul Aziz N., Liew K., Teow S.Y., Abdul Razak F.R., Chin Y.M., Mohamed Shahrehan N.A., Chu T.L., Mohd Kornain N.K. (2017). CD24, CD44 and EpCAM enrich for tumour-initiating cells in a newly established patient-derived xenograft of nasopharyngeal carcinoma. Sci. Rep..

[123] Feng W., Dean D.C., Hornicek F.J., Shi H., Duan Z. (2019). Exosomes promote pre-metastatic niche formation in ovarian cancer. Mol. Cancer.

[124] Guo Y., Ji X., Liu J., Fan D., Zhou Q., Chen C., Wang W., Wang G., Wang H., Yuan W. (2019). Effects of exosomes on pre-metastatic niche formation in tumors. Mol. Cancer.

[125] Liu Y., Cao X. (2016). Characteristics and Significance of the Pre-metastatic Niche. Cancer Cell.

[126] Lane R., Simon T., Vintu M., Solkin B., Koch B., Stewart N., Benstead-Hume G., Pearl F.M.G., Critchley G., Stebbing J. (2019). Cell-derived extracellular vesicles can be used as a biomarker reservoir for glioblastoma tumor subtyping. Commun. Biol..

[127] Mu W., Xu Y., Gu P., Wang W., Li J., Ge Y., Wang H. (2020). Exosomal CD44 cooperates with integrin α6β4 to support organotropic metastasis via regulating tumor cell motility and target host cell activation. Engineering.

